# ScRNA-Seq Analyses Define the Role of GATA3 in iNKT Cell Effector Lineage Differentiation

**DOI:** 10.3390/cells13121073

**Published:** 2024-06-20

**Authors:** Tzong-Shyuan Tai, Huang-Yu Yang, Wan-Chu Chuang, Yu-Wen Huang, I-Cheng Ho, Ching-Chung Tsai, Ya-Ting Chuang

**Affiliations:** 1 Department of Medical Research and Development, Chang Gung Memorial Hospital, Taoyuan 33305, Taiwan; imb503@cgmh.org.tw; 2 Kidney Research Center, Department of Nephrology, Chang Gung Memorial Hospital, Taoyuan 33305, Taiwan; hyyang01@gmail.com; 3 College of Medicine, Chang Gung University, Taoyuan 33302, Taiwan; 4 Advanced Immunology Laboratory, Chang Gung Memorial Hospital, Taoyuan 33305, Taiwan; 5 Department of Medical Research, National Taiwan University Hospital, Taipei 10002, Taiwan; 6 Division of Rheumatology, Inflammation, and Immunity, Department of Medicine, Brigham and Women’s Hospital, 60 Fenwood Road, Boston, MA 02115, USA; iho@bwh.harvard.ed; 7 Harvard Medical School, 60 Fenwood Road, Boston, MA 02115, USA; 8 Department of Pediatrics, E-Da Hospital, I-Shou University, Kaohsiung City 82445, Taiwan; 9 School of Medicine for International Students, College of Medicine, I-Shou University, Kaohsiung City 82445, Taiwan

**Keywords:** iNKT cells, single-cell RNA sequencing, GATA-3, Icos, CD127

## Abstract

While the transcription factor GATA-3 is well-established for its crucial role in T cell development, its specific influence on invariant natural killer T (iNKT) cells remains relatively unexplored. Using flow cytometry and single-cell transcriptomic analysis, we demonstrated that GATA-3 deficiency in mice leads to the absence of iNKT2 and iNKT17 cell subsets, as well as an altered distribution of iNKT1 cells. Thymic iNKT cells lacking GATA-3 exhibited diminished expression of PLZF and T-bet, key transcription factors involved in iNKT cell differentiation, and reduced production of Th2, Th17, and cytotoxic effector molecules. Single-cell transcriptomics revealed a comprehensive absence of iNKT17 cells, a substantial reduction in iNKT2 cells, and an increase in iNKT1 cells in GATA-3-deficient thymi. Differential expression analysis highlighted the regulatory role of GATA-3 in T cell activation signaling and altered expression of genes critical for iNKT cell differentiation, such as *Icos*, *Cd127*, *Eomes*, and *Zbtb16*. Notably, restoration of Icos, but not Cd127, expression could rescue iNKT cell development in GATA-3-deficient mice. In conclusion, our study demonstrates the pivotal role of GATA-3 in orchestrating iNKT cell effector lineage differentiation through the regulation of T cell activation pathways and Icos expression, providing insights into the molecular mechanisms governing iNKT cell development and function.

## 1. Introduction

Invariant natural killer T (iNKT) cells are T cells of innate lineage, characterized by the expression of a highly restricted repertoire of invariant TCRα chains (Vα14-Jα18 in mice and Vα24-Jα18 in humans) coupled with a limited repertoire of Vβ chains (Vβ8, Vβ7, and Vβ2 in mice and Vβ11 in humans). They respond to glycolipid antigens presented by the MHC class I-like molecule CD1d. The iNKT antigen receptor is most effectively bound and thoroughly examined by α-galactosyl ceramide (α-GalCer), a sphingolipid initially discovered in 1994 from the marine sponge Agelas mauritianas. Therefore, iNKT cells can be specifically identified by using CD1d tetramers loaded with α-GalCer. Upon activation, iNKT cells rapidly secrete a large amount of Th1 and Th2 cytokines and chemokines, which then activate DCs, macrophages, NK cells, T cells, and B cells, and drive the progression of adaptive immunity [1]. iNKT cells also express markers of NK cells, producing perforin and granzyme B [2,3,4]. iNKT cells can be further categorized into CD4^+^ and double-negative (DN, CD4^−^CD8^−^) subsets, and a small subset of human iNKT cells are CD8^+^. The CD4^+^ iNKT cell subset has been shown to differ from the DN iNKT cell subset in that CD4^+^ iNKT cells produce greater amounts of IL-4, IL-13, and GM-CSF (Th2 cytokines), whereas DN iNKT cells exhibit greater cytotoxicity and produce more IFNγ and TNF-α [5,6]. Furthermore, recent studies showed that the DN iNKT cell subset also includes iNKT cells that produce IL-17 and IL-22 [7,8]. Despite their small fraction, iNKT cells play pivotal roles in diverse immune responses and diseases, such as tumor surveillance, defense against microbial infection, and the pathogenesis of autoimmune diseases, graft-versus-host disease, obesity, and asthma [9,10,11,12,13,14,15,16].

Similar to conventional T cells, iNKT cells undergo thymic development, transitioning through distinct stages marked by CD24, CD44, and NK1.1 expression. These include immature stage 0 (CD24^+^CD44^−^NK1.1^−^), to transitional stage 1 (CD24^−^ CD44^−^NK1.1^−^), to stage 2 (CD24^−^CD44^+^NK1.1^−^), and finally to mature stage 3 (CD24^−^CD44^+^NK1.1^+^). iNKT cells can achieve functional maturity in the thymus before migrating to peripheral tissues, with emigration occurring mainly at stage 2 and progression to stage 3 in the periphery. Some mature thymic iNKT cells also migrate to the periphery, while others remain as long-term thymus-resident cells. However, this categorization is not ideal, as NK1.1 is not universally expressed in all mouse strains [17,18]. A more recent classification based on PLZF, T-bet, and RORγt aligns with the cytokines IFNγ, IL-4, and IL-17, defining iNKT1, iNKT2, and iNKT17 cells. In this manner, iNKT cells can be defined as PLZF^lo^T-bet^+^RORγt^−^ iNKT1 (IFNγ^+^), PLZF^hi^T-bet^−^RORγt^−^ iNKT2 (IL-4^+^), and PLZF^int^T-bet^−^RORγt^+^ iNKT17 (IL-17^+^) cells. This classification offers a robust foundation for distinguishing iNKT cells based on their functional characteristics. Typically, iNKT1 cells fall under the stage 3 population, iNKT2 cells are present in both the stage 1 and stage 2 populations, and iNKT17 cells are situated within the stage 2 population [19]. Despite these advances, the origins and developmental signals of subsets in the thymus remain unclear, necessitating further research to elucidate their distinct developmental pathways and origins.

The GATA-3 transcription factor, belonging to the C2C2-type zinc finger GATA family, is a crucial regulator in hematopoietic cells, specifically within the T cell lineage. Its significance in thymocyte development and its crucial role in the differentiation of peripheral Th2 cells have been thoroughly characterized. In the absence of GATA-3, the development of CD4SP thymocytes was nearly eliminated, but the generation of CD8SP thymocytes remained unaffected [20,21]. While expressed in both CD4+ and DN iNKT cells, the exact role of GATA-3 in iNKT cell development and function remains less explored. Previous research has indicated that GATA-3 deficiency selectively affects the CD4+ subset of iNKT cells both in the thymus and the periphery. A noteworthy finding is that a considerable portion of GATA-3-deficient iNKT cells express NK1.1 before the up-regulation of CD44, suggesting a potential bypass of stage 2 [22]. However, a comprehensive examination of the distinct factors affecting the development of the iNKT cell subset in the thymus is still lacking and requires further exploration.

In this study, we employed single-cell RNA sequencing to explore the specific role of GATA-3 in iNKT cell development. GATA-3-deficient iNKT cells manifest a complex phenotype, marked by a significant reduction in iNKT2 and iNKT17 subsets, despite seemingly normal numbers of NKT1 cells in the thymus. As a consequence, iNKT1 cells exhibit diminished functionality in the periphery, highlighting the critical role of GATA-3 in the development and/or survival of all iNKT sublineages. These findings contribute to a deeper understanding of the intricate regulatory mechanisms involving GATA-3 in iNKT cell biology.

## 2. Material and Methods

### 2.1. Mice

GATA-3 knockout mice of C57BL/6 genetic background have been described previously [22]. All animals were housed under specific pathogen-free conditions, and experiments were performed in accordance with the institutional guidelines for animal care at the National Taiwan University under approved protocols.

### 2.2. Single-Cell RNA Sequencing and Data Processing

Thymic TCRβ+PBS57-loaded CD1d Tetramer+ iNKT cells were sorted with FACSAria and subjected to scRNA-seq transcriptomic analysis using the 10× Genomics single-cell RNA-seq platform (10x Genomics, Pleasanton, CA, USA). The CellRanger toolkit (version 3.1.0) from 10× Genomics was employed for read alignment and the generation of a gene–cell unique molecule identifier (UMI) matrix. The mouse reference genome mm10 was utilized for gene mapping. The scRNA-seq data were analyzed using the Seurat package (version 5.0.1) in R (Version 4.3.2). The gene counts matrix was loaded into Seurat, and cells that expressed more than 200 genes and genes that were expressed in at least three cells were filtered for further analysis. We also filtered cells that showed unique feature counts over 4000 or less than 200. The doublet cells were filtered by the DoubletFinder package (version 2.0.4) in R. Gene expression was normalized using the SCT-transform. The samples were merged and integrated with the top 3000 variable genes. Cell clusters were generated using 30 PCs at a resolution of one. UMAP dimensional reduction was utilized to visualize the clusters. Trajectory inference was conducted using the Partition-based Graph Abstraction (PAGA) algorithm, a feature-rich tool integrated within Scanpy (version 1.9.6). Differentially expressed genes (DEGs) between FF and FFcre were explored using the function in Scanpy. DEGs were used for GSEA performed using the ClusterProfiler package (version 4.10.0) in R.

### 2.3. FACS Analysis and Antibodies

The following antibody clones were purchased from Biolegend (Biolegend, San Diego, CA, USA) and used for cell surface staining: CD4 (RM4-5), TCR-β (H57-597), NK1.1 (PK136), CD44 (IM7), CD24 (M1/69), CD127 (A7R34), CD69 (H1.2F3), and CD25 (PC61). For nuclear protein staining, cells were fixed and permeabilized using the FOXP3 transcription factor staining kit (Thermo Fisher Scientific, Waltham, MA, USA), according to the manufacturer’s instructions. The following antibodies were used: RORγt (AFKJS-9) was purchased from BD, Ki67 (16A8) and PLZF (Mags.21F7) were purchased from Biolegend, and T-bet (4B10) was purchased from Thermo Fisher Scientific. Flow cytometry was performed on a FACSCanto II or FACSLSRII (BD Bioscience, San Jose, CA, USA) and analyzed with FlowJo software(version 10.10.0). CD1d tetramer, either loaded or unloaded with αGC, was obtained from the NIH Tetramer Facility.

### 2.4. Bone Marrow Transfer

Donor GATA-3 FFcre mice received 150 mg/kg 5-FU 96 h prior to bone marrow harvests. Sca1-positive bone marrow cells were stimulated with mIL-3 (20 ng/mL), mFlt3L (50 ng/mL), hTOP (50 ng/mL), and mSCF (50 ng/mL) for 48 h and spin infected with GFP/CD127-expressing or GFP/ICOS-expressing bi-cistronic retrovirus. At 48 h after transduction, viable cells were harvested and resuspended in sterile PBS at 1 × 10^7^ cells/mL. GFP+ cells were sorted with FACSAria. Recipient congenic CD45.1 mice were irradiated (95 Gy) and received 5 × 10^5^ donor GFP+ cells injected by I.V. administration. Analysis of GFP-positive cells were performed by flow cytometry at 8 weeks post-transplant. The thymic and splenic iNKT cells were determined by flow cytometric analysis using antibodies including CD1d PBS-57 tetramer (NIH Tetramer Core Facility, Atlanta, GA, USA), TCR-β, CD45.2, CD45.1, CD24, CD44, NK1.1, CD4, PLZF, RORγt, and T-bet.

### 2.5. Statistical Analysis

Statistical analyses were performed with Student’s *t*-test unless indicated otherwise. The abbreviation “ns” stands for not significant.

## 3. Results

### 3.1. GATA-3 Deficiency Impairs the Generation of Both iNKT2 and iNKT17 Cells

**Figure 1:**
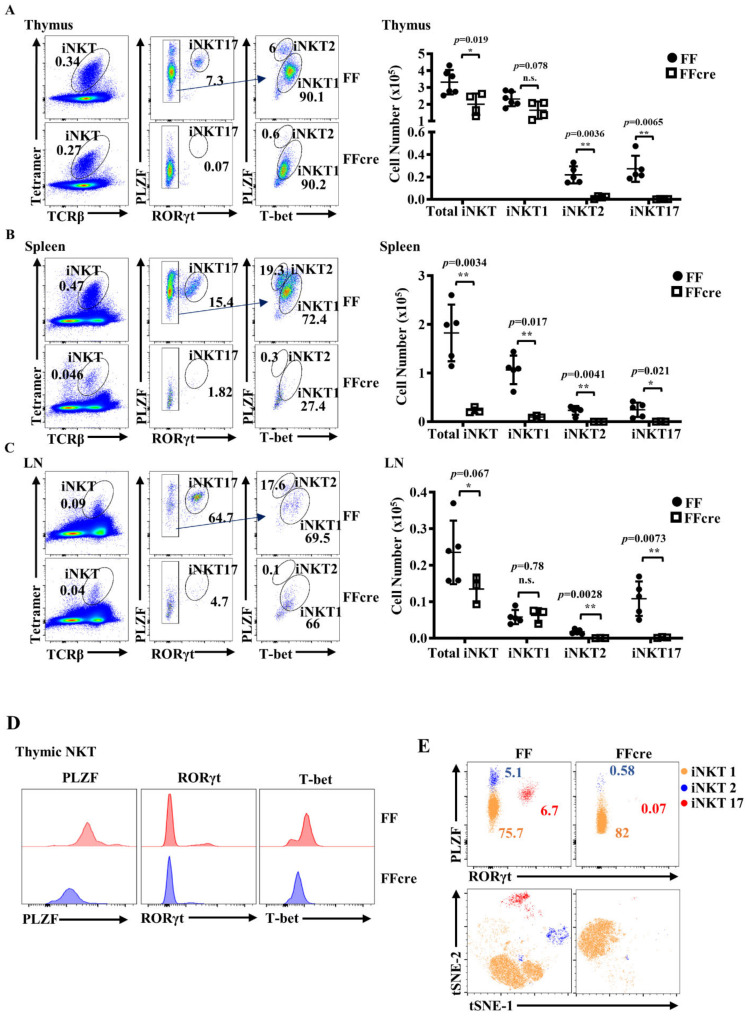
Characterization of iNKT cell effector subsets in GATA-3 FF and FFcre mice. iNKT cells of indicated genotypes in the thymus (**A**), spleen (**B**), and lymph node (**C**) were identified with TCR and loaded CD1d tetramer, and then further divided into iNKT1, iNKT2, and iNKT17 sublineages based on the expression of PLZF, RORγt, and T-bet. The numbers of total iNKT cells and iNKT1/iNKT2/iNKT17 subsets from thymus, spleen and lymph nodes of *GATA-3fl/fl* (FF) and *GATA-3fl/fl Cd4-cre* mice (FFcre) were calculated and statistical analyses were performed with Student’s two-tailed *t*-test. (**D**) The expression levels of PLZF, RORγt, and T-bet of GATA-3 FF and FFcre thymic iNKT cells were quantified with FACS and are shown by histogram. (**E**) Representative conventional bivariate dot plots and t-distributed stochastic neighbor embedding (t-SNE) analysis of iNKT1, iNKT2, and iNKT17 subsets gated by PLZF, RORγt, and T-bet expression of indicated genotypes in the thymus are shown.

We aimed to examine the impact of GATA-3 on the development of iNKT cell effector function. *Cd4-Cre* mice were bred with *GATA-3fl/fl* mice, resulting in the deletion of the GATA-3 gene at the double-positive (DP) developmental stage in the thymus, a crucial phase during which iNKT cells undergo positive selection. To further investigate the generation and distribution of each functionally distinct iNKT subset in *GATA-3fl/fl Cd4-cre* mice, we conducted multicolor flow cytometry analysis on thymic, splenic, and lymph node iNKT cells from *GATA-3 fl/fl* (FF) and *GATA-3fl/fl Cd4-cre* (FFcre) mice to distinguish iNKT1/2/17 sublineage populations. Surprisingly, we noted a near absence not only of iNKT2 cells but also of iNKT17 cells in FFcre mice in the thymus, spleen, and lymph nodes (LNs) (Figure 1A–C). A more modest reduction in iNKT1 cell numbers was also evident in the spleens but not in the thymus and lymph nodes of FFcre mice. Furthermore, the expression levels of the key transcriptional regulators PLZF and T-bet were diminished in thymic iNKT cells from FFcre mice compared to FF control mice (Figure 1D). Collectively, these findings implicate GATA-3 as a critical regulator of PLZF expression in iNKT cells and suggest that the absence of GATA-3 leads to aberrant effector lineage differentiation within the iNKT cell compartment.

To further illustrate the alterations in iNKT1, iNKT2, and iNKT17 populations between FF and FFcre mice, we utilized the dimensionality reduction algorithm, t-distributed stochastic neighbor embedding (t-SNE), for cytometric analysis of the iNKT population. This approach allows for the visualization of single-cell data and qualitative assessment of cell population diversity using 10 parameters, including TCRβ, αGC-loaded CD1d tetramer, CD4, CD24, CD44, NK1.1, PLZF, RORγt, T-bet, and Ki67. The iNKT2 and iNKT17 populations were nearly absent in the thymus of FFcre mice. While the iNKT1 population could still be found in the FFcre thymus, they were predominantly located in a different sub-cluster compared to the FF control, as revealed by t-SNE analysis (Figure 1E). Cells within this sub-cluster exhibited reduced expression of PLZF and CD44, but not T-bet or RORγt, suggesting an immature iNKT1 phenotype (Supplementary Figure S1).

**Figure 2:**
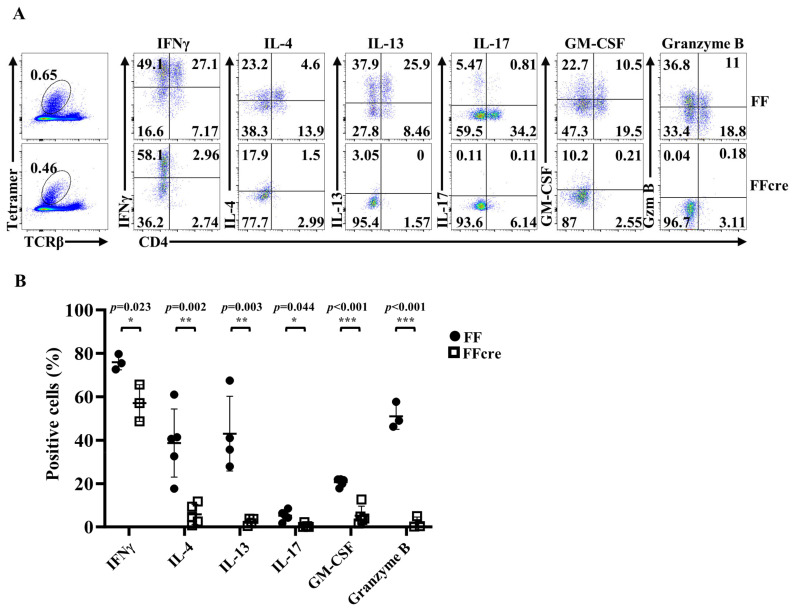
The iNKT effector lineage differentiation in GATA-3 FFcre mice is altered. Thymocytes of FF and FFcre mice were stimulated for 2 h in vitro with PMA and ionomycin. The production of IFNγ, IL-4, IL-13, IL-17, GM-CSF, and Granzyme B by thymic iNKT cells was then analyzed with intracellular cytokine staining. Representative FACS plots are shown in (**A**). Cumulative data from at least 3 mice are shown in (**B**).

To further investigate this observation, we assessed the in vitro effector function of GATA-3-deficient thymic iNKT cells. Intracellular cytokine staining was performed to examine cytokine production by thymic iNKT cells in response to PMA/ionomycin. As expected, Th2- and Th17-related cytokines, such as IL-4, IL-13, and IL-17, were significantly decreased in thymic iNKT cells from FFcre mice. Additionally, FFcre thymic iNKT cells demonstrated reduced production of granzyme B, an iNKT1-associated gene (Figure 2A,B). This observation corresponds to the absence of iNKT2 and iNKT17 cells, along with the presence of a distinct iNKT1 sub-cluster in the GATA-3-deficient thymus (Figure 1).

### 3.2. scRNA-seq Transcriptomic Profiles of the Thymic iNKT Cells from GATA-3 FF and FFcre Mice

**Figure 3:**
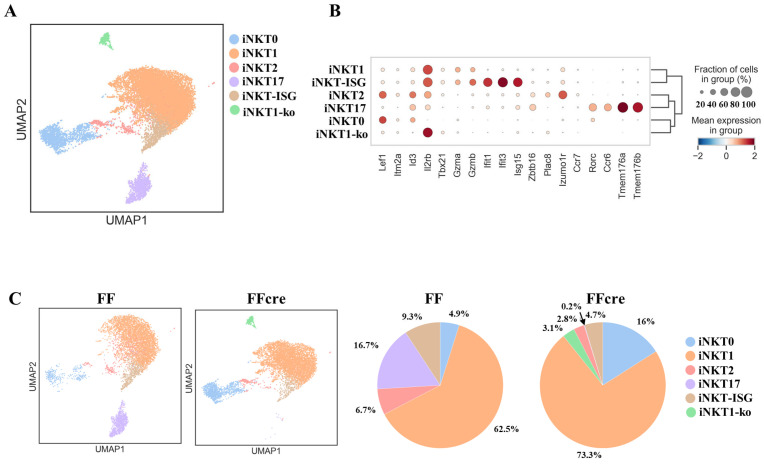
Single-cell RNA sequencing revealed the difference in functional subsets between GATA-3 FF and FFcre iNKT cells. (**A**) Thymic iNKT cells from GATA-3 FF and FFcre mice were sorted and subjected to RNA-based next-generation sequencing (RNA-seq). Transcriptomic analysis was performed using the 10× Genomics platform. Uniform Manifold Approximation and Projection (UMAP) dimensionality reduction analysis showed six major clusters. (**B**) The dot plot shows the expression and distribution of selected genes across the iNKT cell subpopulation. (**C**) UMAP plots (**left panel**) depict the distribution of iNKT subsets from FF and FFcre, while corresponding percentages are displayed in the (**right panel**).

To delve deeper into the role of GATA-3 in iNKT cell development, the thymic TCRβ^+^PBS57-loaded CD1d Tetramer^+^ iNKT cells were sorted and subjected to scRNA-seq (single-cell RNA sequencing) transcriptomic analysis using the 10× Genomics single-cell RNA-seq platform. After quality control, a total of 6498 and 5918 iNKT cells from FF and FFcre samples, respectively, were retained for further analysis, with the exclusion of doublet cells. To gain insight into subpopulation structures, we employed unsupervised graph-based UMAP (Uniform Manifold Approximation and Projection) and clustering through the Louvain algorithm, resulting in the identification of 29 distinct clusters (Supplementary Figure S2). The expression of *Lef1*, *Itm2a*, *Id3*, *Il2rb*, *Tbx21* (encoding T-bet), *Ifit1*, *Ifit3*, *Isg15*, *Zbtb16* (encoding PLZF), *Plac8*, *Izumo1r*, *Ccr7*, *Rorc* (encoding Rorγt), *Ccr6*, *Tmem176a*, and *Tmem176b* were used to define immature populations and effector subsets (Figure 3B and Supplementary Figure S2). We observed that iNKT17 cells (*Rorc+*, *Ccr6+*, *Tmem176a+*) were exclusively present in clusters 1 and 28, while iNKT2 cells (*Zbtb16+*, *Plac8+Izmo1r+*) were identified in clusters 19 and 20. The earliest iNKT precursor cells, iNKT0 cells (*Lef1+Id3+*), were assigned to clusters 0 and 14. The remaining clusters were populated by iNKT1 cells characterized by the expression of *Il2rb* and *Tbx21* (Figure 3A,B and Supplementary Figure S2) [23]. After cluster annotation, we observed a complete absence of iNKT17 cells in FFcre mice. Additionally, there was a substantial reduction in the percentage of iNKT2 cells in FFcre mice, with levels dropping from 6.7% in FF mice to 2.8% in FFcre mice. Conversely, iNKT1 cells showed an increase in FFcre mice, accompanied by a decrease in iNKT-ISG cells (Figure 3C). We identified a uniquely activated iNKT subset in FFcre mice, labeled as iNKT1-ko cells due to their distinct transcriptional profile. These cells exhibited abnormal co-expression of activation markers (high *Zeb2*, *Crtam*) alongside typical iNKT1 genes like *Il2rb*, *Klrb1c*, and *Klre1* [24,25] (Supplementary Figure S3). Unlike conventional iNKT1 cells that follow programmed activation patterns, iNKT1-ko cells exhibited an atypical hyperactivated phenotype uncoupled from normal cellular activation processes. The presence of this dysregulated subset exclusively in FFcre mice suggests a disruption in the regulation of homeostatic signals governing the balanced activation and functional differentiation of iNKT cells.

**Figure 4:**
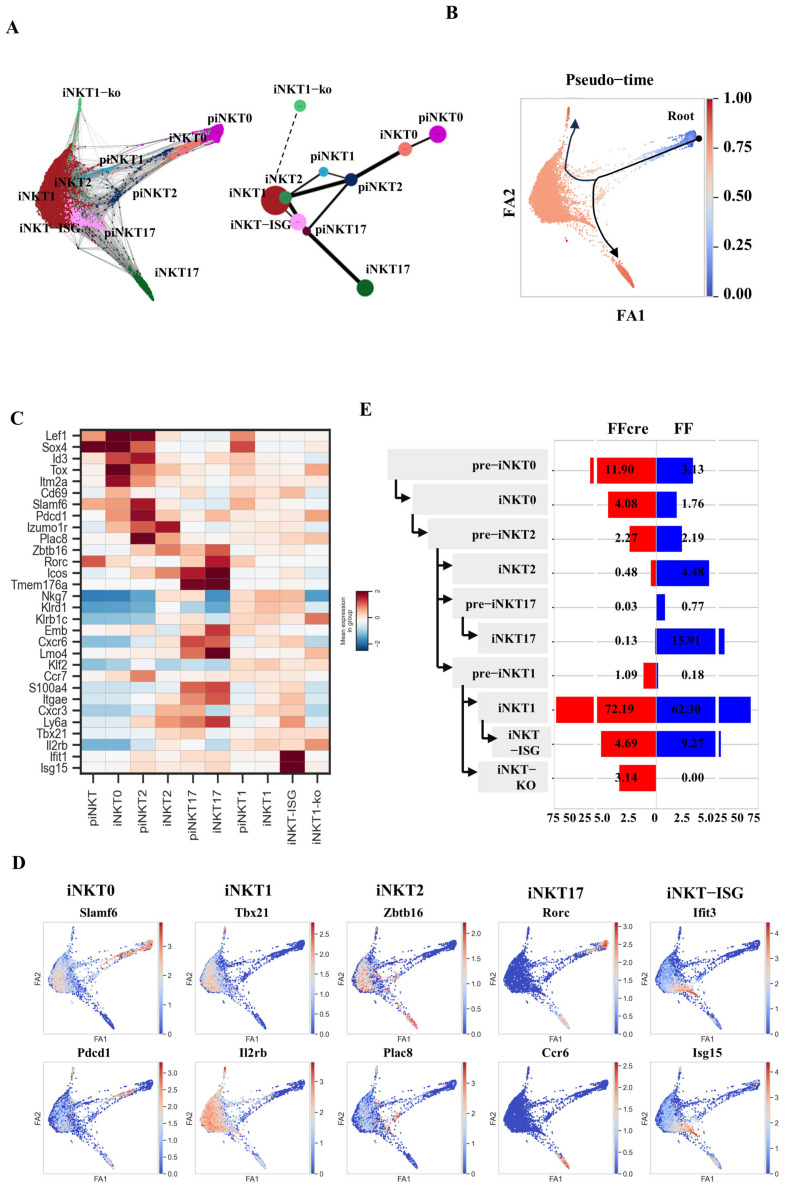
Predictive developmental trajectories of iNKT thymocytes in GATA3 FF and FFcre mice. (**A**) Partition-based Graph Abstraction (PAGA) analysis was applied to the single-cell RNA sequencing data obtained from GATA-3 FF and FFcre iNKT thymocytes. The PAGA graph (**right panel**) and PAGA-initialized single-cell embedding (**left panel**) are presented. Weighted edges in the graph represent a statistical measure of connectivity between clusters. (**B**) Pseudo-time analysis was conducted to delineate the developmental trajectory of iNKT cells, with piNKT0 serving as the Root point. (**C**) The expression patterns of a representative list of cell-subset-specific gene markers are depicted. (**D**) Visualization of selected markers of PAGA-initialized single-cell embedding alongside the trajectories defined in (**A**). (**E**) The percentage of cell subsets in FF and FFcre mice is illustrated, with deduced developmental pathways indicated by arrows.

To delve into the differentiation paths of iNKT cell subsets, we conducted further characterization of the clusters and generated a graph-based map using Partition-based Graph Abstraction (PAGA) analysis (Figure 4A). This map illustrates the interconnection between clusters and organizes them along potential differentiation routes [26]. In the analysis, we tried to pinpoint cells in the initial phases of differentiation within each iNKT subset, which we denoted as pre-iNKT0. For example, pre-iNKT0 cells exhibited diminished expression of lymphocyte activation genes (e.g., *Itm2a*, *Cd69*) and iNKT-associated transcription factors (*Lef1*, *Sox4*, *Id3*, and *Tox*) compared to mature iNKT0 cells. Similarly, pre-iNKT1 cells showed reduced expression of iNKT1 signature genes (e.g., *Nkg7*, *Klrd1*) relative to iNKT1 cells, indicating a precursor phenotype. Intriguingly, pre-iNKT2 cells expressed the T cell zone homing receptor *Ccr7*, resembling precursor iNKT cells reported previously [27]. Additionally, pre-iNKT2 also expressed genes associated with iNKT17 differentiation like *Emb* and *Cxcr6*, suggesting a potential for plasticity towards an iNKT17 fate. The pre-iNKT1 cells exhibited an intermediate state between the pre-iNKT2 cells (expressing *Izumo1r* and *Plac8*) and the iNKT1 cells (expressing *Tbx21* and *Il2rb*). This was based on their co-expression of genes characteristic of both subsets, suggesting that iNKT1 cells could arise from the differentiation of pre-iNKT2 cells. Additionally, the persistent expression of certain iNKT1 gene markers (e.g., *Il2rb*, *Tbx21*) in iNKT2 cells indicated their potential to further differentiate along the iNKT1 lineage, similar to the recently identified iNKT2b population [28] (Figure 4A). Collectively, these findings pointed to the existence of multiple developmental pathways leading to the generation of iNKT1 cells.

Utilizing the expression of early iNKT selection markers like *Slamf6* and *Pdcd1*, we designated pre-iNKT0 cells as the starting point for pseudo-time ordering (Figure 4B,D). The inferred model indicated that pre-iNKT0 cells progress through iNKT0 to pre-iNKT2 cells along the PAGA trajectories and pseudo-time ordering (Figure 4B,D). This transition was characterized by robust induction of *Izumo1r* and *Zbtb16* [29] (Figure 4D). Subsequently, the pseudo-time path branched into three distinct trajectories, giving rise to iNKT17, iNKT1, or iNKT1 subsets. The iNKT17 pathway showed the induction of crucial genes such as *Rorc* and *Tmem176a* (Figure 4B,D). The iNKT1 trajectory was characterized by up-regulation of signature genes, including *Il2rb* and *Tbx21*. Finally, a subset of cells expressed elevated levels of interferon-stimulated genes (ISGs) like *Ifit1* and *Isg15*, representing an iNKT-ISG population analogous to those reported recently [28,30] (Figure 4B,D).

Upon analysis of the percentage of each iNKT cell population and their pseudo-time development trajectory, we observed an increase in pre-iNKT0 and iNKT0 stages in FFcre mice compared to FF mice. Although the percentage of pre-iNKT2 cells was similar between the two groups, the subsequent iNKT2 and pre-iNKT17 populations were found to be decreased in FFcre mice. In contrast, a slight increase in the pre-iNKT1 cell population was observed in FFcre mice (Figure 4E). These findings suggest that GATA-3 deficiency may impede the flexibility of pre-iNKT2 to differentiate into either iNKT2 or pre-iNKT17, thereby inhibiting the generation of both iNKT2 and iNKT17 cells.

### 3.3. GATA-3 Controls iNKT Cell Activation Signal

**Figure 5:**
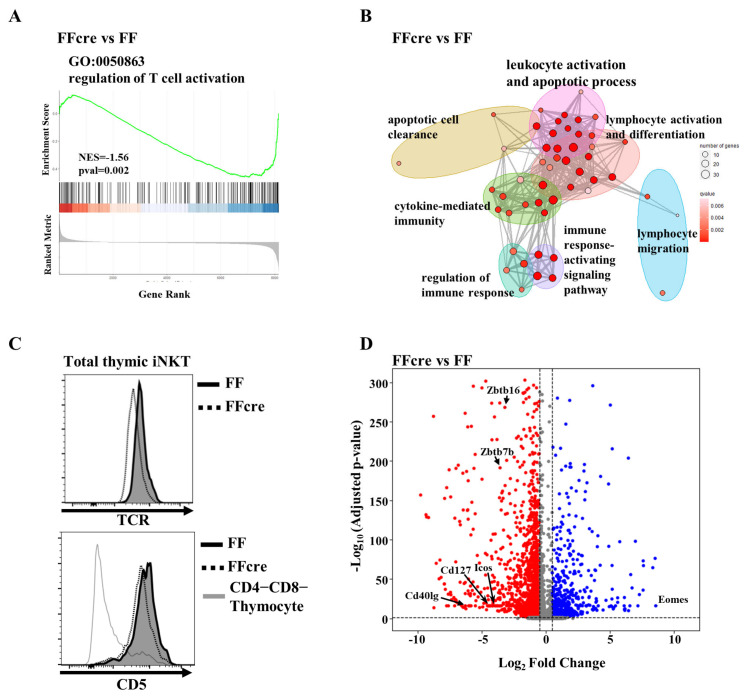
The activation signal within iNKT cells is attenuated in GATA-3 FFcre mice. (**A**) Gene Set Enrichment Analysis (GSEA) results revealed the downregulation of genes associated with the T cell receptor signaling pathway in GATA-3 FFcre iNKT cells. (**B**) The enrichment map illustrates the significantly suppressed functions in FFcre compared to FF iNKT cells. Nodes represent GO gene sets, while edges indicate shared genes between GO gene sets. Node size corresponds to the number of perturbed genes within a given gene set. (**C**) Total thymic iNKT cells from GATA-3 FF and FFcre mice exhibit differential expression of TCR (T cell receptor) and CD5. (**D**) The volcano plot highlights genes involved in the T cell receptor signaling pathway that show differential expression between GATA-3 FF and FFcre iNKT cells.

A proposed model of iNKT cell differentiation posits that strong T cell receptor (TCR) signaling promotes the iNKT17 lineage, moderate signaling favors the iNKT2 fate, and weak signaling skews development toward the iNKT1 subset. Corroborating this model, previous studies have demonstrated that iNKT17 cells exhibit the highest TCR expression levels compared to iNKT2 and iNKT1 cells [31]. Notably, defective TCR signaling has been associated with the loss of iNKT2 and iNKT17 cells [32], reminiscent of the phenotype observed in GATA-3-deficient mice. Gene Set Enrichment Analysis (GSEA) of our transcriptomic data revealed dysregulation of T cell activation pathways in FFcre iNKT cells (Figure 5A). Gene set overrepresentation analysis also identified significant enrichment of differentially expressed genes (DEGs) involved in lymphocyte and leukocyte activation pathways (Figure 5B). In addition, we observed downregulated TCR and CD5 expression, which is correlated with TCR signaling strength [33,34], in FFcre iNKT cells compared to FF controls (Figure 5C). Consistent with these findings, we identified several genes associated with T cell activation, such as *Zbtb7b* (Supplementary Figure S4), *Cd127*, *Icos*, *Cd44*, *Cd40lg*, and *Plzf*, whose expression was attenuated in the absence of GATA-3. Along with previous studies showing that CD25, CD69, and CD40L expression are lower in FFcre iNKT cells upon stimulation, this suggests that the defect in TCR expression translates to a functional defect. Collectively, our results suggest that the absence of iNKT2 and iNKT17 cells in GATA-3-deficient mice may be partially attributable to perturbations in TCR signaling.

### 3.4. GATA-3 Regulates Gene Expression in Various Subsets

**Figure 6:**
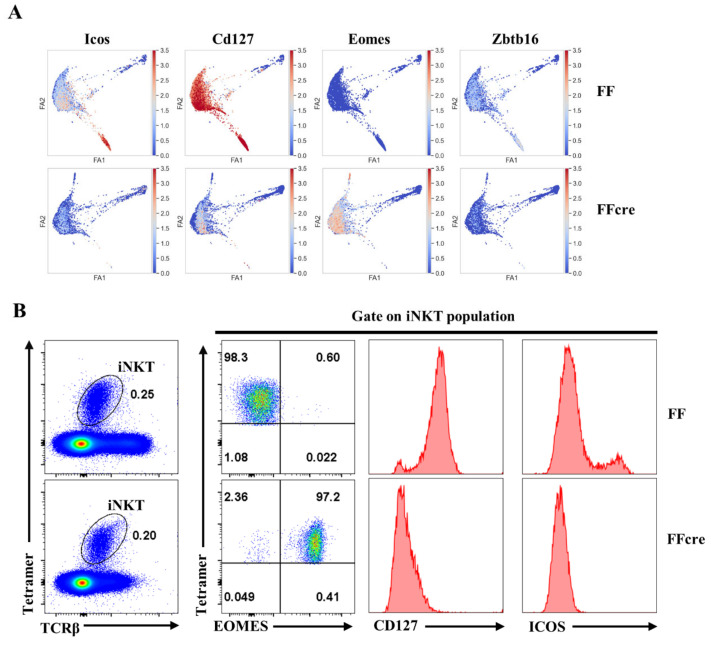
Surface expression of CD127 and ICOS in different cell types of GATA-3 FF and FFcre mice. (**A**) The expression and distribution patterns of Icos, Cd127, Eomes, and Zbtb16 genes are depicted using single-cell RNA sequencing data. (**B**) Representative flow cytometry plots of iNKT cells gated from FF and FFcre thymocytes, depicting the expression level of EOMES, CD127, and ICOS. Results are representative of three independent experiments.

We further investigated differentially expressed T cell activation-associated genes between FF and FFcre iNKT cells that could potentially impact iNKT cell differentiation. *Icos* gene is predominantly expressed in iNKT17, with some expression in iNKT1, and its expression level was downregulated in FFcre iNKT cells. *Cd127* also exhibits high expression in both iNKT17 and iNKT1, with the expression level being downregulated in FFcre iNKT cells. *Eomes* is primarily seen in iNKT1, but in FFcre, the expression of *Eomes* was higher in both iNKT1 and iNKT2 (Figure 5D and Figure 6A). *Zbtb16* is mainly expressed in all iNKT cells except iNKT0, and its expression in FFcre iNKT cells was lower across all examined iNKT cell subsets (Figure 6A). To assess the protein expression levels and validate the scRNA data of the differentially expressed genes, we utilized flow cytometry. Our analysis demonstrated an up-regulation of EOMES expression in FFcre thymic iNKT cells, coupled with a notable decrease in the expression of CD127 and ICOS (Figure 6B). These findings suggest that GATA-3 may contribute to the regulation of iNKT effector lineage differentiation through various factors, such as PLZF, CD127, and ICOS.

### 3.5. GATA-3 Affects iNKT2 and iNKT17 Differentiation by Regulating CD127 or ICOS

**Figure 7:**
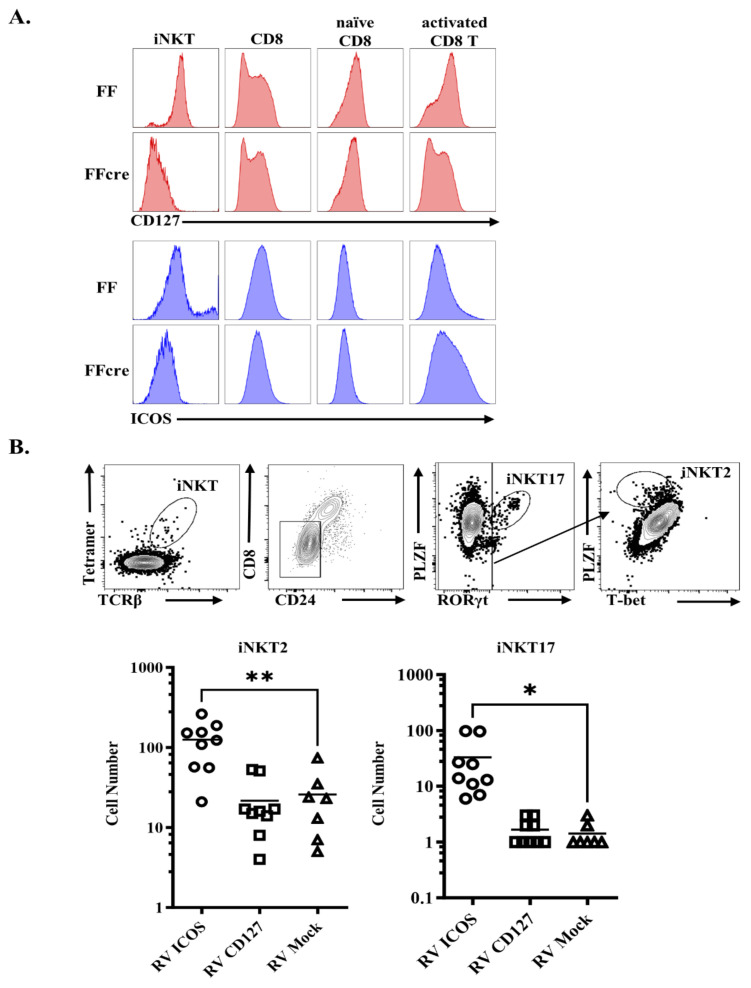
Overexpression of ICOS rescues iNKT2 and iNKT17 cells of GATA-3-deficient mice. (**A**) The thymocytes and splenocytes from GATA-3 FF and FFcre mice were stained with TCRβ, CD4, CD8, CD44, CD62L, NK1.1, loaded CD1d tetramer, CD127, and ICOS. The histogram plots show the surface expression levels of CD127 and ICOS on thymic iNKT cells (TCRβ^+^ tetramer^+^), CD8 single-positive thymocyte (TCRβ^hi^CD4^−^CD8^+^), splenic naïve (TCRβ^+^CD4^−^CD8^+^CD44^−^CD62L^+^), and activated/memory (TCRβ^+^CD4^−^CD8^+^CD44^hi^CD62L^−^) CD8 T cells. (**B**) The gating strategy for identifying iNKT17 and iNKT2 cells from FFcre thymocytes overexpressing ICOS and CD127 via retroviral transduction is illustrated. Cumulative results are presented in the (**lower panel**). (two-sided unpaired *t*-test, ns, * *p* < 0.05, ** *p* < 0.01).

Moreover, we also found GATA-3 controls CD127 and ICOS expression in a cell-type-specific manner. The decreased expression of CD127 and ICOS is more obvious in FFcre iNKT cells, compared with either CD8 single-positive thymocytes or splenic naive CD8 T cells (Figure 7A). We further investigated whether attenuated expression of CD127 and ICOS can explain the iNKT cell effector lineage differentiation defects caused by GATA-3 deficiency in vivo. We infected FFcre bone marrow cells (CD45.2) with a GFP/ICOS- or GFP/CD127-expressing bi-cistronic retrovirus to restore ICOS or CD127 expression (Supplementary Figure S5). Subsequently, the transduced cells were sorted and transplanted into irradiated CD45.1 WT mice, and the transduced CD45.2+ FFcre iNKT cells in host animals were identified by the co-expression of GFP. The iNKT cell subsets were further characterized as tetramer-positive cells and by their expression of the transcription factors PLZF, RORγt, and t-BET (Figure 7B). Interestingly, the restoration of ICOS, but not CD127, consistently increased the cell numbers of iNKT2 and iNKT17 cells in the thymus (Figure 7B). The data indicate that GATA3 controls iNKT cell development through the regulation of ICOS gene expression.

## 4. Discussion

Invariant natural killer T (iNKT) cells, a unique subset of T cells, hold a crucial role in various immune responses. Previous studies have linked the intrinsic regulation of iNKT cells to the transcription factor GATA-3, known for its significance in the development and function of conventional CD4 Th cells. Our current study provided an in-depth analysis of how GATA-3 influences the differentiation of functional subsets within iNKT cells. Our data unequivocally demonstrated that GATA-3 deficiency impairs the generation of both iNKT2 and iNKT17 cells. Intracellular staining revealed a pronounced downregulation in the expression levels of PLZF and RORγt in GATA-3-deficient thymic iNKT cells. By employing single-cell RNA sequencing (scRNA-seq) analysis, we identified several intriguing genes exhibiting differential expression between GATA-3 FF and FFcre samples across each iNKT subpopulation, including CD127 and ICOS. Notably, our findings highlight that the overexpression of ICOS, but not CD127, can restore iNKT cell development.

It is noteworthy that GATA-3-deficient thymic iNKT cells predominantly manifest an iNKT1 phenotype. Despite expressing the invariant Vα14Jα18 TCR and producing the Th1-related cytokine IFNγ, these residual thymic iNKT1 cells exhibit significantly lower T-bet expression compared to iNKT1 cells from the FF control mice (Figure 2). Furthermore, GATA-3 FFcre thymic iNKT cells fail to produce granzyme B (Figure 2), a hallmark gene enriched in iNKT1 cells. These findings shed light on the nuanced role of GATA-3 in shaping the functional diversity of iNKT cell subsets.

The results of our differentiation trajectory analysis suggest a sequential development pattern, indicating that iNKT0 cells initially differentiate into iNKT2 cells, followed by maturation leading to the emergence of iNKT17 cells, as evidenced by the trajectory’s endpoint (Figure 4A,B). Furthermore, our dendrogram analysis, consistent with prior studies [28], highlights a notable similarity in gene expression profiles between iNKT2 and iNKT17 cells (Figure 3B and Supplementary Figure S2), strongly implying a potential linkage in their developmental pathways. Importantly, our data propose a plausible explanation for the observed decrease of iNKT17 cells in GATA-3-deficient mice. It appears that this reduction in iNKT17 cell population correlates with a corresponding decrease within the iNKT2 cell population. This insight deepens our comprehension of the interplay between these subsets and elucidates the repercussions of GATA-3 deficiency for the dynamics of iNKT cell differentiation.

CD127, also known as the IL-7 receptor α chain (IL-7Rα), is a type I transmembrane glycoprotein expressed on various immune cells, such as thymocytes (excluding CD4/CD8 double-positive thymocytes), peripheral T cells, and innate lymphoid cells (ILCs). Previous studies have highlighted the critical role of GATA3 in the development of all IL-7Rα-expressing ILCs [35]. Additionally, Zhong et al. demonstrated that GATA-3 influences ILC3 homeostasis by modulating IL-7Rα [36]. In the context of iNKT cells, previous research indicates that the homeostasis of IL-17-producing RORγt+ iNKT cells relies on IL-7 rather than IL-15. Notably, exposure to IL-7 prompts rapid cellular proliferation in iNKT17 cells, independent of TCR–CD1d contact [37]. However, our rescue experiment yielded intriguing results, showing that the overexpression of CD127 had no discernible effect on iNKT cell development. This observation suggests that, in a GATA-3-deficient setting, CD127 alone is not sufficient for the proper development of iNKT cells.

On the contrary, ICOS (CD278), recognized as an inducible costimulatory molecule, is typically expressed on activated CD4+ T cells post-activation. However, it exhibits a notably high level of expression on iNKT cells, even in their resting state [36]. Previous studies have elucidated that the interaction between ICOS on iNKT cells and its ligand on dendritic cells or B cells is not obligatory for the differentiation and maturation of iNKT cells. Instead, ICOS plays a crucial role in the maintenance of peripheral CD4+ iNKT cells. Deficiency in ICOS has been associated with a notable reduction in CD4+ iNKT cells in peripheral organs [38,39], resembling the phenotype observed in our GATA-3 FFcre mice. Interestingly, our data indicate that among multiple cell types, ICOS expression is substantially higher in iNKT cells, and the absence of GATA-3 completely abolishes its expression (Figure 6A), emphasizing its predominant role in iNKT cells. Upon the overexpression of ICOS in GATA-3-deficient mice, a significant increase in iNKT cells is observed in both the thymus and spleen, further supporting the notion that ICOS functions downstream of GATA-3 in regulating iNKT cell development (Figure 7B).

Our study provides insights for manipulating the ICOS signaling pathway to influence the differentiation of iNKT cell subsets. iNKT cells exhibit significantly reduced immunogenicity compared to other peripheral blood-derived immune cells like αβ T cells, γδ T cells, and natural killer cells, rendering them promising candidates for off-the-shelf, cell-based cancer immunotherapies. With the diverse subsets within the iNKT cell population, a better understanding of the mechanisms driving the anti-tumor functions of various human iNKT cell subpopulations is crucial for achieving significant advances in their exploitation for cancer therapies, potentially leading to improved clinical efficacy [40]. Recent advances in hematopoietic stem cell (HSC) gene engineering and in vitro differentiation have led to the development of human allogeneic HSC-engineered iNKT (AlloHSC-iNKT) cells, which demonstrate promising tumor-killing capabilities in animal models [41,42]. AlloHSC-iNKT cells serve as a valuable model for manipulating iNKT cell differentiation and selection, as well as the utilization of specific human iNKT cell subsets to enhance outcomes in iNKT-based cancer immunotherapy.

In summary, our single-cell methodology applied to GATA-3-deficient mice revealed a dynamic sequence of transcriptional events, underscoring the central role of GATA-3 in steering the development of iNKT cells, particularly at a crucial branch point in this process. This comprehensive analysis provides insights into the specific genes impacted along this developmental pathway, highlighting them as potential key contributors to iNKT cell biology.

## 5. Limitations of Current Study

While the findings reported in this study provide valuable insights into the role of GATA3 in iNKT cell effector lineage differentiation, there are several limitations and considerations that should be acknowledged. First, investigating the alterations found in a disease model where iNKT cells play a relevant role would further strengthen the implications of our findings. Exploring the effects of GATA3 deficiency in an appropriate animal disease model could elucidate the functional consequences of the observed alterations in a clinically relevant context. Second, analyzing the cytokine response in vivo, such as α-GalCer stimulation, would confirm the reported findings [22] and provide additional functional validation. In vivo stimulation experiments could corroborate the proposed role of GATA3 in modulating iNKT cell effector functions and cytokine production. Third, while our study highlights the importance of ICOS as a downstream target of GATA3, it is important to consider that several other co-stimulatory molecules may be involved in mediating the effects of GATA3 on iNKT cell differentiation and function. Further investigations are needed to identify and characterize additional co-stimulatory molecules and pathways regulated by GATA3 in this context. Addressing these limitations in future studies will provide a more comprehensive understanding of the role of GATA3 in iNKT cell biology and its potential implications for disease processes.

## Data Availability

The original contributions presented in this study are included in the article/Supplementary Materials. Further inquiries can be directed to the corresponding author.

## Supplementary Materials

The following supporting information can be downloaded at: https://www.mdpi.com/article/10.3390/cells13121073/s1, Figure S1: Differential gene expression between FF and FFcre iNKT1 cells. Figure S2: Expression of Signature genes in indicated cell type. Figure S3: Heatmap displaying the expression levels of the indicated genes across different iNKT cell subsets. Figure S4: Violin plot showing the expression levels of Zbtb7b across different iNKT cell subsets. Figure S5: Retroviral transduction of CD127 and ICOS in Sca1+ bone marrow cells.
