# Supplementary material for: ScRNA-Seq Analyses Define the Role of GATA3 in iNKT Cell Effector Lineage Differentiation
Source: Cells. 2024 Jun 20;13(12):1073. doi: 10.3390/cells13121073 (PMC11201670; doi:10.3390/cells13121073)
Supplement: Supplementary file 1 [file cells-13-01073-s001.zip › cells-3044601-supplementary.pdf]

Supplementary data

ScRNA-seq Analyses Define the Role of GATA3 in iNKT Cell Effector Lineage Differentiation

Tzong-Shyuan Tai, Huang-Yu Yang, Wan-Chu Chuang, Yu-Wen Huang, I-Cheng Ho, Ching-Chung Tsai, Ya-Ting Chuang

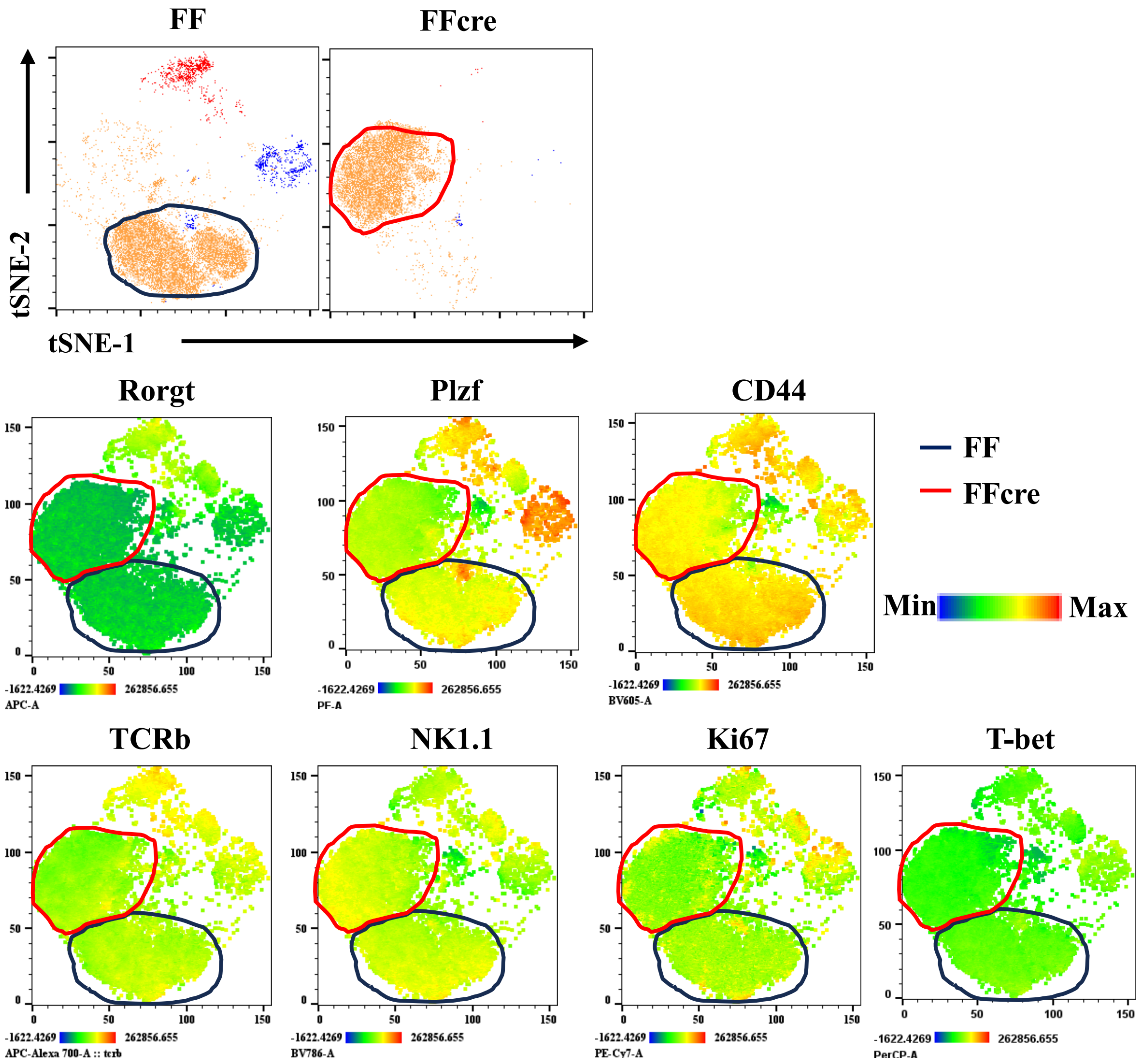

Supplementary Figure S1 Differential gene expression between FF and FFcre iNKT1 cells.
Heatmaps showing the expression levels of the indicated genes are displayed.

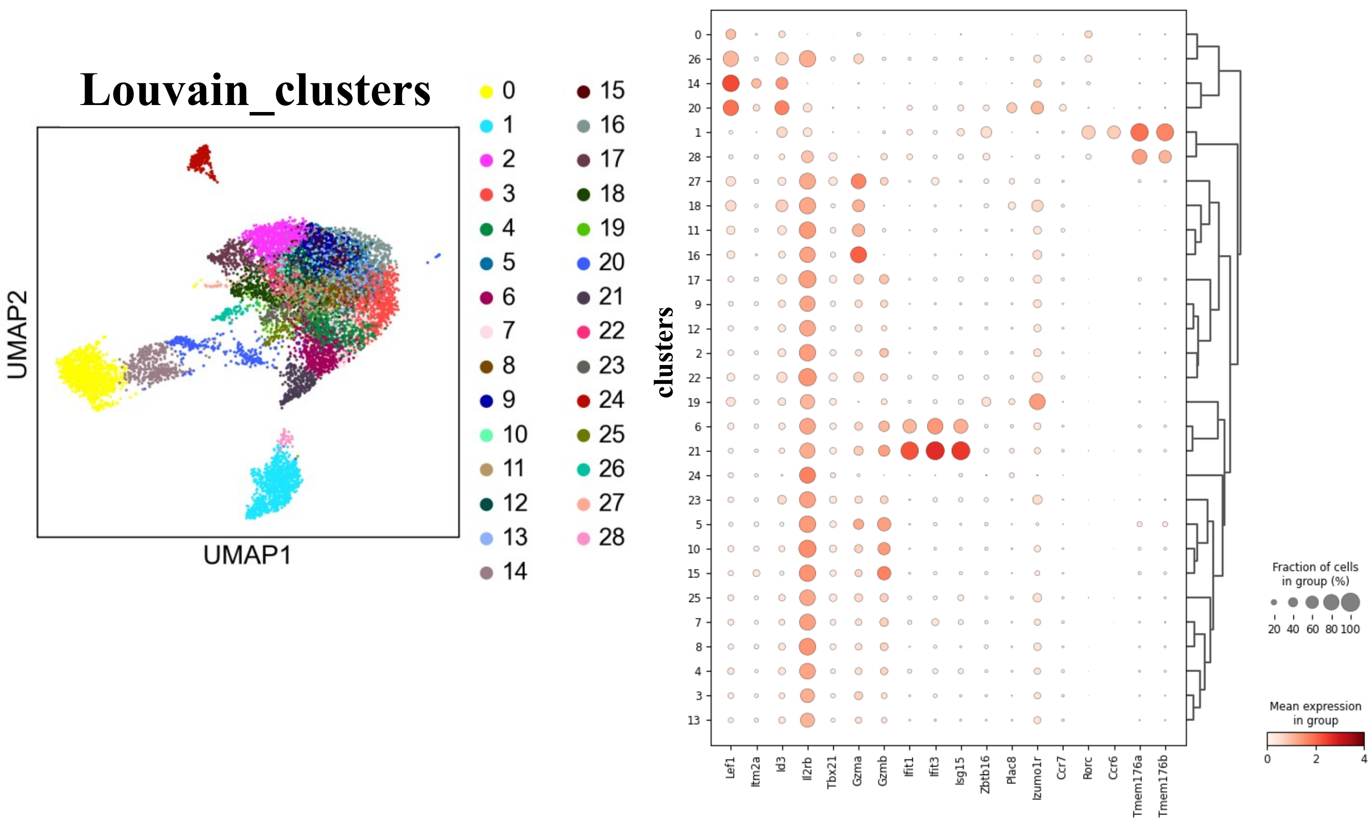

Supplementary Figure S2 Expression of Signature genes in indicated cell type

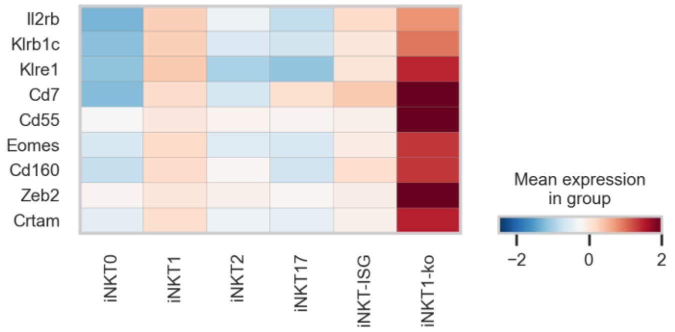

Supplementary Figure S3 Heatmap displaying the expression levels of the indicated genes across different iNKT cell subsets.

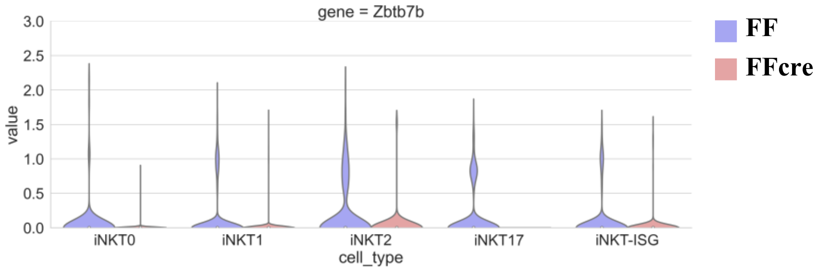

Supplementary Figure S4 Violin plot showing the expression levels of Zbtb7b across different iNKT cell subsets.

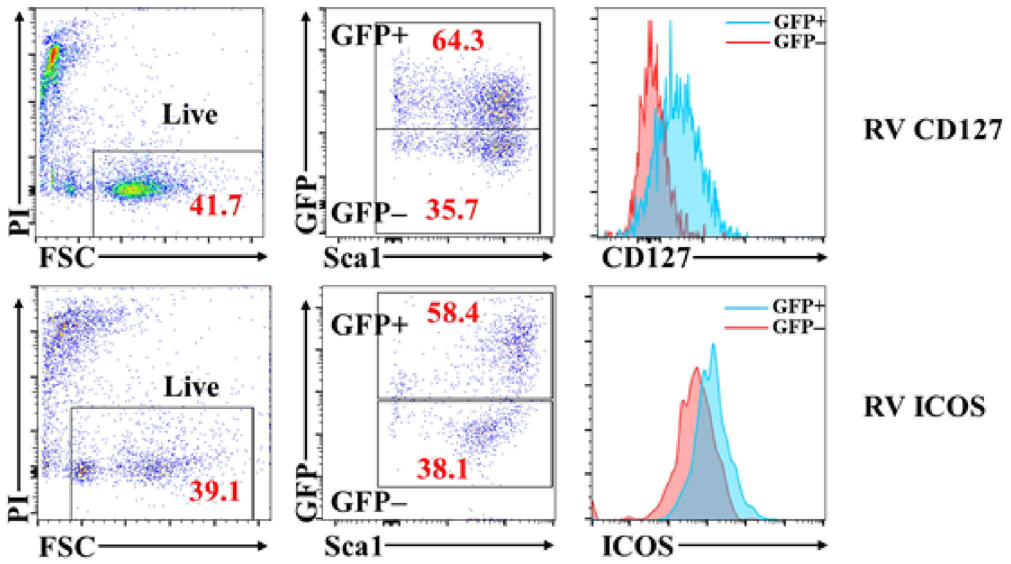

Supplementary Figure S5 Retroviral transduction of CD127 and ICOS in Sca1+ bone marrow cells. ScaI positive bone marrow cells were stimulated with mIL-3 (20 ng/ml), mFlt3L (50 ng/ml), hTOP (50 ng/ml), and mSCF (50 ng/ml) for 48 hours and spin infected with GFP/CD127 or GFP/ICOS bi-cistronic retrovirus.
